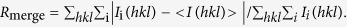# Structural and Physical Basis for Anti-IgE Therapy

**DOI:** 10.1038/srep11581

**Published:** 2015-06-26

**Authors:** Jon D. Wright, Hsing-Mao Chu, Chun-Hsiang Huang, Che Ma, Tse Wen Chang, Carmay Lim

**Affiliations:** 1 Institute of Biomedical Sciences, Academia Sinica, Taipei 115, Taiwan; 2 The Genomics Research Center, Academia Sinica 115, Taiwan; 3 Department of Chemistry, National Tsing Hua University, Hsinchu 300, Taiwan

## Abstract

Omalizumab, an anti-IgE antibody, used to treat severe allergic asthma and chronic idiopathic urticaria, binds to IgE in blood or membrane-bound on B lymphocytes but not to IgE bound to its high (FcεRI) or low (CD23) affinity receptor. Mutagenesis studies indicate overlapping FcεRI and omalizumab-binding sites in the Cε3 domain, but crystallographic studies show FcεRI and CD23-binding sites that are far apart, so how can omalizumab block IgE from binding both receptors? We report a 2.42-Å omalizumab-Fab structure, a docked IgE-Fc/omalizumab-Fab structure consistent with available experimental data, and the free energy contributions of IgE residues to binding omalizumab, CD23, and FcεRI. These results provide a structural and physical basis as to why omalizumab cannot bind receptor-bound IgE and why omalizumab-bound IgE cannot bind to CD23/FcεRI. They reveal the key IgE residues and their roles in binding omalizumab, CD23, and FcεRI.

Most prevalent allergic diseases, e.g., allergic asthma, allergic rhinitis, atopic dermatitis, and food allergy, are caused by immunoglobulin E (IgE) mediated type-I hypersensitivity reactions. IgE is responsible for allergic reaction caused by exposure to allergens such as dust mites, pollen, mold, animal dander, and peanuts. It mediates an allergic reaction *via* interaction with its two receptors, high-affinity FcεRI on mast cells and basophils^1^ and low-affinity CD23 on B cells. Free soluble IgE binds to FcεRI on the surface of mast cells, basophils, and antigen-presenting dendritic cells. Binding of soluble CD23 to membrane-bound IgE and the complement receptor CD21 on B cells results in an increased production of IgE (Fig. 1). In a sensitized individual, allergens bind to allergen-specific IgE and cross-link the IgE/FcεRI complexes, triggering the release of pharmacological and inflammatory mediators, causing various allergic symptoms.

Because IgE is a key mediator in allergic reactions, one way to treat IgE-mediated allergic diseases is to target both membrane-bound and soluble IgE^2^. Such an approach is advantageous as it is independent of allergens. Furthermore, IgE is early in the allergic pathway and appears to be dispensable^3^. Indeed, a humanized anti-IgE antibody called omalizumab (trade name Xolair) has been developed to target the IgE pathway and has successfully undergone or is being studied in 136 clinical trials (see www.clinicaltrials.gov). Omalizumab has been approved for treating not only patients with severe, persistent allergic asthma, but also patients with recalcitrant, antihistamine-resistant chronic idiopathic urticaria^4^,^5^,^6^. It has been studied in combination with allergen-based specific immunotherapy (allergy shots) to (i) reduce anaphylactic reactions when receiving allergen immunizations and (ii) accelerate immunization schedule and dosing to achieve faster therapeutic effects in more patients. The success of omalizumab in treating patients with asthma has clarified dispute whether IgE plays a role in the pathogenesis and symptom manifestation of asthma.

What differentiates the therapeutic omalizumab from an ordinary anti-IgE? An ordinary anti-IgE can cross-link FcεRI-bound IgE and aggregate FcεRI. If it were injected into a person, it could cause massive activation and degranulation of mast cells and basophils, leading to anaphylactic shock and possible death. In contrast to an ordinary anti-IgE, the therapeutic omalizumab does *not* bind IgE already bound by FcεRI or CD23 on the cell surface or soluble CD23 in blood, but it can still bind to membrane-bound and soluble IgE^2^,^7^. Such a therapeutic anti-IgE averts the anaphylactic effects exhibited by an ordinary anti-IgE because by binding to soluble IgE, omalizumab blocks the interaction between IgE and its receptors, depleting both free and receptor-bound IgE. By depleting IgE, omalizumab indirectly decreases FcεRI density on basophils and antigen-presenting cells^8^,^9^,^10^ (as IgE-free FcεRI is structurally unstable and becomes internalized and degraded), thus reducing mast cell/basophil activation and antigen presentation to T cells. Furthermore, omalizumab forms small immune complexes with IgE^11^, whose fragment antigen-binding (Fab) regions remain free to bind allergens; thus these immune complexes serve as “antigen-sweepers^12^”.

How does omalizumab block IgE from binding to both CD23 and FcεRI? An early model structure of IgE in complex with CGP56901^11^, the first anti-IgE developed in 1988 with the aforementioned specificities, indicates that the binding sites for CGP56901 and FcεRI overlap^13^. Subsequent site-directed mutagenesis studies^14^ confirm that some of the IgE residues implicated in binding omalizumab are located in the FcεRI-binding site. X-ray structures of the IgE Cε3 and Cε4 (abbreviated as Cε3-4) domains in complex with FcεRI^15^,^16^ and CD23^17^,^18^ show that the CD23 and FcεRI-binding sites on IgE do not overlap and are in fact far apart: the FcεRI-binding site is near the N-termini of both Cε3 domains, whereas the CD23-binding site is at the opposite end, near the Cε3-4 junction. Although crystal structures of two different anti-IgE Fab bound to IgE have been reported, neither shares high sequence identity with omalizumab^19^,^20^. Hence, the IgE residues crucial for binding omalizumab are unknown, so how omalizumab prevents IgE from binding to both its receptors remains puzzling. Moreover, since IgE has two identical heavy chains, it is not clear why omalizumab cannot bind to the FcεRI-free chain or why FcεRI cannot bind to the free Cε3 domain in the 1:1 IgE/omalizumab complex.

Interestingly, the X-ray structures of IgE free and bound to its receptors or anti-IgE show different conformations (orientations) for the Cε2 and Cε3 domains relative to the Cε3 and Cε4 domains, respectively. Relative to the Cε4 domains, the Cε3 domains are “closed” when bound to the CD23 but are “open” when bound to FcεRI (Fig. 2, top). The closed Cε3-Cε4 conformation seen bound to CD23 cannot bind to FcεRI^17^, but can bind to omalizumab^21^, whereas the open Cε3-Cε4 conformation seen bound to FcεRI cannot bind to CD23^17^. In contrast to the receptor-bound IgE structures, the *free* IgE structure (PDB 2wqr) shows an open Cε3-Cε4 conformation in one chain and a closed one in the other, while the Cε2 domain pair folds back against the Cε3 domains (Fig. 2, bottom). This bend of the Cε2 domain relative to Cε3 is apparently unaffected by binding to CD23^22^, but is enhanced by binding to FcεRI^16^, and becomes unbent upon binding to an anti-IgE antibody^20^ (Fig. 2, bottom).

Since the early IgE/CGP56901 model^11^,^13^, several developments and experimental data enable a reliable prediction of the IgE/omalizumab structure. Several structures of IgE domains have been solved including crystal structures of IgE-Fc with the Cε2 domains in a bent conformation (PDB 2wqr) and captured in an extended conformation (PDB 4j4p). Individual components of the binding free energy have been used to train a support vector machine (SVM) classifier to detect native conformations among the thousands of refined antibody/antigen (Ab/Ag) conformations^23^. Tests on 24 Ab/Ag complexes from the protein-protein docking benchmark version 3.0 showed that in each test case, a SVM classifier could rank the native conformation in the top ten among the thousands of refined Ab/Ag conformations^23^. These top-ranking conformations could then be screened for one that is most consistent with experimental data.

Here, we have solved the crystal structure of the omalizumab-Fab and docked it to the free IgE-Fc X-ray structure using our SVM classifier to obtain a complex structure consistent with known experimental data (see Results). Based on ensembles of conformations generated from molecular dynamics (MD) simulations of the Cε3-4 dimer in complex with omalizumab-Fv, CD23, and FcεRI in explicit water, we have computed the binding free energy contributions of each residue. The MD structures and per-residue free energies reveal the key IgE residues involved in binding omalizumab, FcεRI, and CD23. They provide a physical basis for the set of unique binding specificities of omalizumab, the understanding of which has hitherto been elusive.

## Results

The IgE residue numbers employed follow the KABAT numbering^24^, which is not contiguous. To distinguish omalizumab residues from IgE ones, the former will be preceded by the complementarity-determining region (CDR) loop; e.g., L3:His92 indicates His92 from the CDR-L3 loop.

### The crystal structure of omalizumab Fab

The 2.42-Å crystal structure of the omalizumab-Fab region shows a highly negatively charged surface (see Fig. 3a, which shows the surface electrostatic potentials from the APBS program^25^ for the six CDRs). The L1, L2, and L3 CDRs exhibit negative surface potentials, whereas the heavy chain CDR loops are neutral. Surprisingly, the three histidines in the H3 loop (His97, His100a, and His100c), which were assumed to be positively charged in previous works^14^,^26^, are predicted to be neutral by five programs (Reduce^27^, Whatif^28^, PDB2PR^29^, PROPKA3^30^, and HAAD^31^). The neutral state of H3:His100c is consistent with its low solvent-accessible surface area (SASA) of 3% in the crystal structure. Although H3:His100a (SASA = 15%) and H3:His97 (SASA = 31%) are partially solvent exposed, they are hydrogen-bonded to each other and well-packed with vdW contacts to nearby hydrophobic residues including H3:His100c (Fig. 3b). The H3, L1, and L3 loops contain residues implicated in direct/indirect binding to IgE from site-directed mutagenesis studies^26^, namely, H3:His97, H3:His100c, L1:Asp30, L3:Glu93, and L3:Asp94.

### The docked IgE-Fc/omalizumab-Fab structure is consistent with available experimental data

To obtain a structure of omalizumab bound to IgE, the crystal structures of omalizumab-Fab and human IgE-Fc (PDB 4gt7 and 4j4p) consisting of the Cε2, Cε3, and Cε4 domains (denoted as Cε2-3-4) were docked together, as described in Methods. The predicted IgE-Fc/omalizumab-Fab structure is consistent with the following experimental data:

1. The docked IgE-Fc/omalizumab-Fab structure shows that L3:Glu93, whose mutation along with L3:Asp94 to Ala reduced binding to IgE^26^, forms a salt bridge with Arg457 in the Cε3 domain.
2. It also shows that all the IgE residues experimentally implicated in binding omalizumab are within 5 Å of the omalizumab residues. These IgE residues are Ser407, Arg408, Ser411, Lys415, Glu452,^455^QCRVT^459^, Arg465, and Met469 whose mutation to Glu407/Gln407, Glu408, Gln411, Asp415, Arg452/Gln452, ^455^ACAVA^459^, Glu465, and Ala469 significantly reduced or nearly abolished binding to omalizumab^14^. They also include the ^462^HLP^464^ motif determined from fine epitope mapping of omalizumab^32^.
3. The orientation of omalizumab-Fab bound to IgE allows for two omalizumabs to bind to a single IgE (Fig. 4a), in agreement with experimental results^33^.
4. The omalizumab-binding site on IgE is near the binding sites for CD23 and FcεRI.
5. The total SASA change upon binding the omalizumab-Fab and IgE-Fc (2,054 Å^2^) is consistent with the buried surface areas (1,144–2,500 Å^2^) computed for 22 Ab/Ag complexes in the protein-protein docking benchmark version 3.0^34^.

### The IgE/omalizumab interface

The IgE-Fc/omalizumab-Fab structure consistent with available experimental data was used as the starting point for four sets of MD simulations in explicit water to create an ensemble of conformations, as described in Methods. These conformations were used to compute average distances between IgE and omalizumab heavy atoms. An interface residue is defined by a mean heavy–heavy atom distance ≤5 Å between IgE and omalizumab. A hydrogen bond is defined by a mean hydrogen–acceptor atom distance ≤2.4 Å and a donor–hydrogen–acceptor angle >130^o^, while a vdW contact is defined by a mean heavy–heavy atom distance ≤4.0 Å at least 50% of the time in two or more simulations.

The omalizumab interface residues in the IgE/omalizumab complex are distributed among all the CDRs except the L2 loop (Supplementary Table S1). Missing from the IgE/omalizumab interface are H3:His100c and L1:Asp30, which have been implicated in binding IgE from mutagenesis studies^26^ (see above). These two omalizumab residues appear to play a conformational role in binding to IgE: In the MD structures, the buried H3:His100c forms vdW contacts with L:Tyr49, H3:Ser96, and H3:Phe99 and a backbone-backbone hydrogen bond to H3:His100a, which in turn is hydrogen-bonded to H3:His97 and H3:Ser96 (Fig. 3b). This well-packed core helps to rationalize why simultaneous mutation of the three His residues in the H3 loop to Ala abolished binding to IgE^26^. The L1:Asp30 side-chain points towards the protein interior in the X-ray structure and is hydrogen-bonded to L1:Ser31, while its backbone is in vdW contact with L1:Tyr27D and L1:Asp28, which binds IgE via hydrogen bonds with Ser471 and Arg470, respectively. Thus, L1:Asp30 plays a role in stabilizing the L1 loop for binding omalizumab, hence, its mutation to Ala would destabilize the L1 loop, accounting for the observed loss of binding to IgE^26^.

The IgE interface residues in the IgE/omalizumab complex stem from two nearly linear epitopes (Supplementary Table 1): The first nearly contiguous sequence involves ^405^T-SR^408^ from β-strand C and ^410^ASGKP^416^ in the following CD loop (there are no residues with KABAT numbers 409, 412, and 413; a dash indicates absence of the residue at the interface). This epitope forms hydrogen bonds or vdW contacts with the L3, H2, and H3 loops. The second nearly contiguous sequence consists of Glu450 from helix-B, ^451^GET^453^ in the EF' turn, ^455^Q-R-T^459^ in β-strand F, ^460^HPHLPRA^466^ in the FG loop, and ^467^LMRS^471^ in β-strand G (there is no residue with KABAT number 468). This epitope forms hydrogen bonds or vdW contacts with the L1, L3 and H2 loops. Although the interface residues are formed from two disparate sequences, they are located together with β-strands C, F, and G forming an anti-parallel β-barrel structure (Fig. 4b). Notably, the interface residues encompass all residues experimentally implicated in binding omalizumab^14^,^32^ (see above).

### Roles of key IgE residues in binding omalizumab

Which residues make the most favorable contributions towards binding omalizumab? To address this question, the binding free energy contribution of each IgE residue was computed using 8,000 conformations sampled from four simulations of the Cε3-4 dimer bound to omalizumab-Fv in explicit water. Although the scheme used to compute the binding free energy cannot yield accurate *absolute* free energies due to the continuum solvent approximation used to compute the interaction free energy^35^ (see Methods), it can yield trends in the *relative* free energy contributions of residues towards binding a given ligand. The free energy contributions of non-interface residues are insignificant, so only those of the interface residues are listed in Supplementary Table 1. Among the IgE interface residues, Ser407, Ala410, Ser411, Lys415, Arg457, Arg465, Met469, and Arg470 make significantly favorable contributions to binding omalizumab (see Fig. 5a). These residues have been implicated in binding omalizumab from site-directed mutagenesis^26^ except Ala410 and Arg470 whose roles in binding omalizumab have not been experimentally examined. The ^462^HLP^464^ motif is also experimentally implicated in binding omalizumab, but its net free energy contribution is relatively small (–2.3 kcal/mol).

Although mutagenesis studies reveal that Ser407, Arg408, Ser411, Lys415, Glu452 ^455^QCRVT^459^, Arg465, and Met469 reduced or nearly abolished omalizumab binding (see above), they cannot discern if these residues directly contact omalizumab or provide some conformational stabilization. The roles of these residues can be deduced from their interactions and free energy contributions towards binding omalizumab: Ser407, Lys415, Arg457, Arg465, and Met469 directly hydrogen bond to omalizumab and make significant binding free energy contributions. On the other hand, Arg408 and Glu452 stabilize the IgE conformation critical for binding, but do not directly contact omalizumab and make negligible (–1 kcal/mol) binding free energy contributions. These two residues are salt-bridged to each other and indirectly bind omalizumab: Arg408 forms a side chain–backbone hydrogen bond with Lys415, which in turn is salt-bridged to H2:Asp54, while Glu452 forms a side chain–side chain hydrogen bond with Ser411, which is in vdW contact with H2:Asn58.

The interactions found are consistent with and help to rationalize the mutagenesis results. For example, the Ser411---Glu452---Arg408---Lys415---H2:Asp54 hydrogen-bonding network found in the MD simulations can explain why mutation of Arg408 to Glu or Glu452 to Arg significantly reduced binding to omalizumab^14^: These mutations result in repulsive Glu408---Glu452 and Arg452---Arg408 interactions, resulting in conformational changes that hinder IgE from binding omalizumab. Likewise, mutation of Ser407, Lys415, and Arg465 to an acidic residue (Glu or Asp) nearly abolished binding to omalizumab^14^, as these three residues interacted with omalizumab Asp residues during the simulations: Ser407 formed transient hydrogen bonds with L3:Asp94, Lys415 is salt-bridged to H2:Asp54, while Arg465 is salt-bridged to L1:Asp27C. The loss of binding to omalizumab upon mutation of ^455^**QCRVT**^459^ to ^455^ACAVA^459^^14^ can be attributed mainly to Arg457, which formed side chain–side chain hydrogen bonds with the L3:His92 and L3:Glu93 in the simulations. On the other hand, the loss of binding to omalizumab upon mutation of Met469 to Ala^14^ is likely due to loss of packing interactions, as the Met469 side chain formed multiple vdW contacts with L1:Tyr27D, L1:Tyr32, and L3:His92 in the simulations. In contrast to Met469, mutation of the neighboring Ser471 to Ala did not significantly affect omalizumab binding^14^, in line with its insignificant free energy contribution (–0.8 ± 0.8 kcal/mol), despite its direct omalizumab contact via a sidechain–sidechain hydrogen bond with L1:Tyr27D.

### Key IgE residues involved in binding the IgE receptors

To determine if the IgE residues involved in binding omalizumab are also crucial for binding CD23 or FcεRI, the free energy contributions from the Cε3-4 residues towards binding either IgE receptor were computed from four independent simulations of the Cε3-4 dimer bound to CD23 or FcεRI (see Methods). The MD simulations could maintain the structural integrity of the IgE/receptor crystal structures: The Whatif program^28^ indicated fourteen potential hydrogen bonds between the IgE and its receptor in the X-ray structure of IgE in complex with CD23 (PDB 4gko)^18^ and nine for FcεRI (PDB 1f6a)^15^. In each case, all but two putative IgE---receptor hydrogen bonds in the crystal structures were preserved in at least two simulations (Supplementary Table S2). The free energy contributions from the IgE interface residues upon binding to CD23 (Fig. 5b) and FcεRI (Fig. 5c) reveal the most important IgE regions involved in binding the IgE receptors and those residues that are shared by the low/high-affinity receptor and omalizumab.

### CD23

The IgE/CD23 interface residues are found mainly in four sequential regions encompassing the Cε3-4 domains from one of the heavy chains (Supplementary Table S3a). Two regions consisting of ^408^**R**A**S**x**K**^415^ and ^446^RDxIEG**E**^452^ contains crucial omalizumab-binding residues (bold), notably Lys415 and Glu452, whose mutations to Asp and Arg, respectively, nearly abolished binding to omalizumab^14^. Lys415 is salt-bridged to CD23:Asp193, while Glu452 is hydrogen-bonded to neutral CD23:His186. Apart from binding CD23, Glu452 also links the two regions containing omalizumab-binding residues via hydrogen bonds with Ser411. These two regions make significantly favorable free energy contributions (–8 and –13 kcal/mol) to binding CD23.

The other CD23-binding regions do not involve omalizumab-binding residues. One of them consists of Lys474 and Ser476 in the Cε3–Cε4 linker followed by ^497^GPRAA^501^ in the Cε4 domain. This region (in particular Lys474, Ser476 and Arg499) makes a large free energy contribution to binding CD23 (–25 kcal/mol) that is comparable to the CD23-binding regions encompassing omalizumab-binding residues (–21 kcal/mol). In contrast, the other “unique” CD23-binding region, which consists of ^592^EAASxSQ^598^ in the Cε4 domain, makes a much smaller binding free energy contribution (–4 kcal/mol). These two CD23-specific regions are linked by salt bridges between Glu592 and Lys474/Arg499.

### FcεRI

Unlike CD23, which binds to residues in the Cε3 and Cε4 domains belonging to the same IgE chain, the FcεRIα domain binds to residues in both Cε3 domains but not to residues in either Cε4 domain (Supplementary Table S3b). The FcεRIα-binding residues are found in four sequential regions, two of which are partially duplicated on the second chain. The two duplicated FcεRIα-binding regions contain residues found at the IgE/omalizumab interface. One of these “duplicated” regions is ^460^H-HL^463^ in chain B and the ^461^PHLPR^465^ motif in chain A. The latter makes a large favorable free energy contribution (–17 kcal/mol) towards binding FcεRIα with Pro464 contributing slightly over half (–9 kcal/mol). The ^462^HLPR^465^ motif has been experimentally implicated in binding omalizumab (see above). Notably, His462, Pro464, and Arg465 form hydrogen bonds with FcεRIα Trp110, Ser85, and Asp86 side chains, respectively. Interestingly, omalizumab seems to mimic the interactions made by FcεRIα with Pro464 and Arg465, as L1:Ser27A and L1:Asp27C hydrogen bond to Pro464 and Arg465, respectively. The other “duplicated” region is the ^365^RGV^367^ motif in chain B, which is contained in the longer ^363^NPRGVA^368^ motif in chain A. These two motifs make significant favorable free energy contributions (–5 and –11 kcal/mol) towards binding FcεRIα with Arg365 making the largest contribution. Pro364 from chain A and Arg365 from chain B are close to omalizumab. The other two FcεRIα-binding regions comprising ^394^DLAPS^398^ and ^428^RNGT^434^ in IgE chain B, which do not involve omalizumab-binding residues, also make quite favorable free energy contributions (–12 and –7 kcal/mol) towards binding FcεRIα.

## Discussion

Although omalizumab was found *via* screening IgE-specific IgG1 antibodies that could not bind to FcεRI-bound IgE^2^,^7^, it was not clear why omalizumab prevents IgE from binding to both receptors (see Introduction). It was also not clear which IgE residues directly bind omalizumab and which provide conformational stability^14^,^26^. By docking the crystal structures of omalizumab-Fab (solved herein) and human IgE-Fc (PDB 4gt7) we have obtained an IgE-Fc/omalizumab-Fab structure consistent with available experimental data, which has hitherto been unsolved. From the binding free energy contributions of each residue, we have elucidated which IgE residues directly bind omalizumab, which play a conformational role, and which are in common for binding omalizumab and the low/high-affinity receptor. These results explain why omalizumab cannot bind receptor-bound IgE and why omalizumab-bound IgE cannot bind to FcεRI or CD23 (see below).

**Figure 2:**
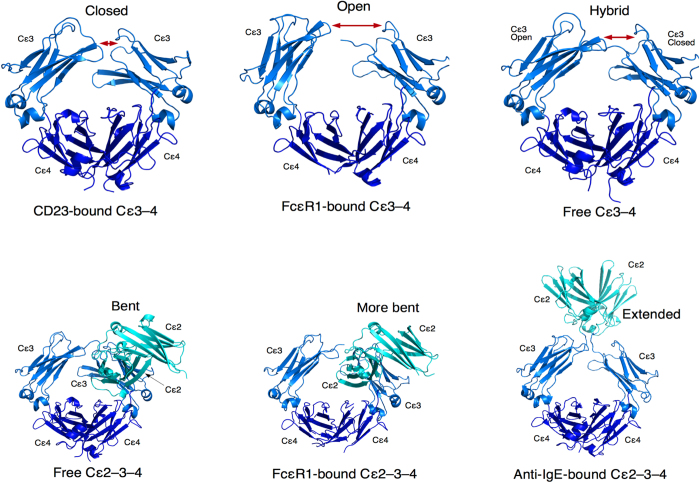
Conformational changes in the IgE-Fc upon binding its receptors. Top: The Cε3 domains adopt a closed conformation when bound to CD23 (PDB 4gko), an open one when bound to FcεRI (PDB 1f6a), and a hybrid conformation with chain A “open” and chain B “closed” when IgE is free in solution (PDB 2wqr). Bottom: The Cε2 domains adopt a bent conformation with contacts to one of the Cε3-4 domains in free IgE (PDB 2wqr), becomes even more bent upon binding FcεRI (PDB 2y2q), but is extended when bound to two non-omalizumab anti-IgE molecules (PDB 4j4p).

**Figure 3:**
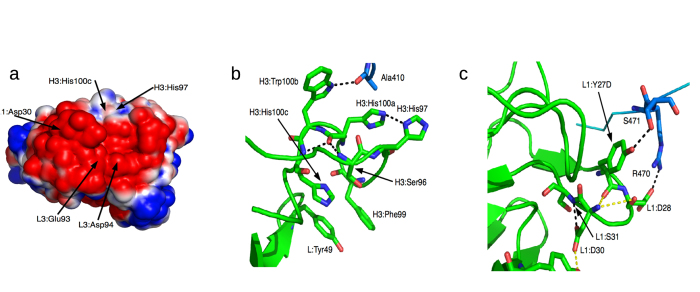
The omalizumab-Fv region. (**a**) Electrostatic potentials derived from the 2.42 Å crystal structure; arrows indicate the residues implicated in binding IgE from site-directed mutagenesis studies. (**b**) Packing and hydrogen-bonding interactions of the three histidines in the H3 loop. (**c**) Interactions of L1:Asp30 showing that its side chain points away from the protein surface. Omalizumab-Fv residues are in green and IgE residues in blue.

**Figure 4:**
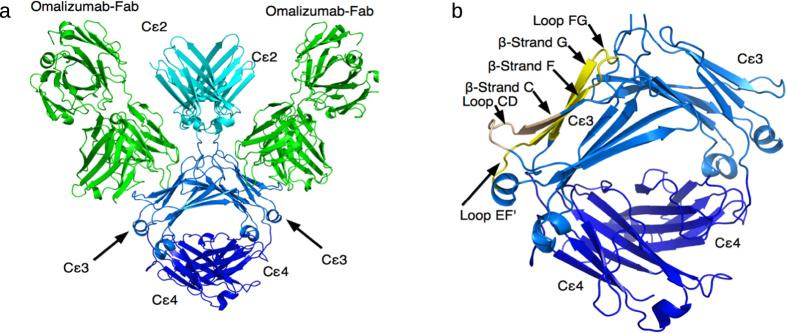
The IgE/omalizumab interface. (**a**) Two omalizumab-Fab molecules (green) binding to two IgE Cε3-4 domains (marine/blue) with the Cε2 domains (cyan) in the extended conformation. (**b**) Omalizumab-binding site (yellow/wheat) on the IgE Cε3 domain.

The IgE/omalizumab-Fab complex structure indicates that (i) the Cε2 domains have to move away from the bent conformation in the free IgE structure to unmask the second omalizumab-binding site and (ii) omalizumab could bind to both open and closed conformations of Cε3-4 domains. Free IgE-Fc in solution is predominantly bent with the Cε2 domains folded back onto the Cε3-4 domain of one chain^36^,^37^,^38^ (Fig. 2, bottom left), but the Cε2 domains can flip to the other Cε3-4 domain, forming another bent conformation via transiently extended conformations^20^ (Fig. 2, bottom right). Free IgE-Fc with predominantly bent or transiently extended Cε2 conformations can bind a single omalizumab (Fig. 6). However, if the IgE-Fc were to remain bent after binding one omalizumab, then the Cε2 domain, which packs against the Cε3 domain, would obstruct binding of a second omalizumab, in conflict with the experimentally observed 1:2 IgE/omalizumab complex^11^,^33^. This is evident in Fig. 6a, where the Cε3 domains in the 1:2 IgE-Fc/omalizumab-Fv complex in Fig. 4a are superimposed onto those in the free IgE crystal structure (PDB 2 wqr). This superposition also shows that omalizumab can bind to both closed and open conformations of the Cε3-4 domains, as long as the Cε2 domains are in an upright position: The two omalizumabs exhibit no clashes with IgE after the Cε3 domains from the 1:1 IgE-Fc/omalizumab-Fv complex were separately superimposed onto the closed and open Cε3-4 conformations of the 2 wqr structure (Fig. 6b).

Omalizumab can compete with the CD23 and FcεRI in binding to IgE, as its affinity for IgE-Fc (K_A_ ~ 10^9^–10^10^ M^−1^)^19^ is comparable to FcεRI (K_A_ ~ 10^8^–10^10^ M^−1^) and much higher than a single CD23 (K_A_ ~ 10^6^–10^7^ M^−1^)^1^. The omalizumab-binding site in the Cε3 domain consists of two distinct sequences: (i) ^405^RxSRASGKP^416^, and (ii) ^450^EGETxQxRxTHPHLPRALMRS^471^. It can form 1:1 and 1:2 complexes with IgE. The binding orientation of omalizumab to IgE and the relative free energy contributions explain how omalizumab inhibits the binding of IgE to both its receptors, resulting in profound effects on the attenuation of IgE-mediated allergic responses.

**Figure 5:**
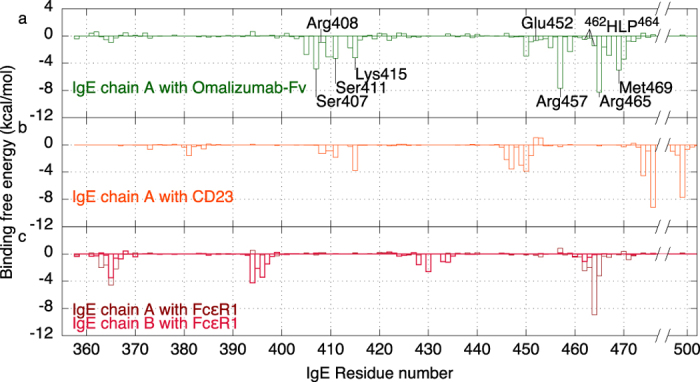
Free energy contributions of IgE residues towards binding (**a**) omalizumab-Fv, (**b**) CD23, and (**c**) FcεRI. Residues experimentally implicated in binding omalizumab are labeled.

**Figure 6:**
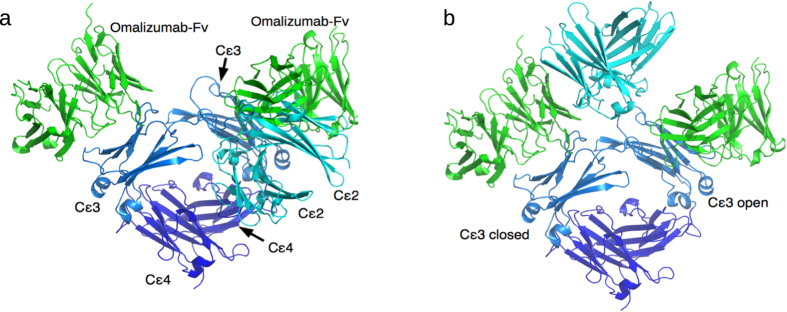
Two omalizumab molecules bind to IgE in an extended Cε2 conformation with the Cε3-Cε4 dimer in a closed or open conformation. (**a**) The Cε2 domains in the free IgE structure clashes with the second omalizumab-Fv domain after superposition of each Cε3 domain (blue) from the 1:2 IgE-Fc/omalizumab-Fv complex onto that from the free IgE structure (PDB 2wqr) and displaying only omalizumab-Fv. (**b**) Both omalizumab-Fv domains exhibit no clashes with IgE after one of the Cε3 domains from the 1:2 IgE-Fc/omalizumab-Fv complex is superimposed onto the closed Cε3 conformation of chain A in PDB 2wqr, whereas the other Cε3 domain is superimposed onto the open Cε3 conformation of chain B; only omalizumab-Fv and the Cε2 domains in the omalizumab–Fab/IgE-Fc complex are displayed, while the Cε2 domains in the free structure are hidden.

**Figure 7:**
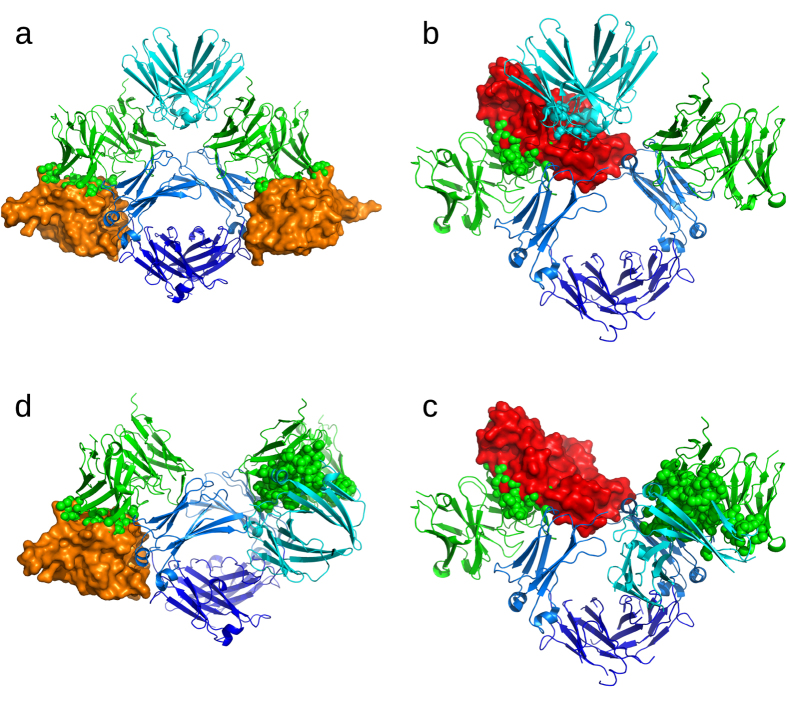
Why omalizumab-bound IgE cannot bind CD23 or FcεRI and why omalizumab cannot bind to receptor-bound IgE. (**a**) CD23 (tangerine) clashes with omalizumab–Fab (green) after the IgE Cε3 domains from omalizumab-bound IgE are separately superimposed onto that from CD23-bound IgE (PDB 4gko), and displaying only the omalizumab-Fv. The IgE-Cε2 domains in the extended position are modeled by superimposing the IgE Cε3-4 domains from PDB 4j4p onto those in CD23-bound IgE and displaying only the IgE Cε2 domains. Omalizumab heavy atoms within 2.5 Å of heavy atoms in CD23 are depicted as spheres. (**b**) FcεRI (scarlet) clashes with the IgE-Cε2 domains (cyan) in the extended position after each IgE Cε3 domain from omalizumab-bound IgE is separately superimposed onto that from FcεRI-bound IgE (PDB 2y7q), and displaying only omalizumab-Fv. The IgE-Cε2 domains were modeled in an extended position by superimposing the IgE-Cε3-4 domains from PDB 4j4p over the FcεRI-bound IgE and displaying only the Cε2 domains in the 4j4p structure, while hiding the IgE-Cε2 domains from 2y7q. Omalizumab and IgE-Cε2 heavy atoms within 2.5 Å of heavy atoms from FcεRI are depicted as spheres. (**c**) FcεRI (scarlet) clashes with omalizumab–Fv (green) after each Cε3 domain from omalizumab-bound IgE is separately superimposed onto that from FcεRI-bound IgE (PDB 2y7q), and displaying only omalizumab-Fv. Omalizumab heavy atoms within 2.5 Å of heavy atoms from FcεRI or IgE-Cε2 are depicted as spheres. (**d**) Omalizumab–Fv (green) clashes with CD23 or the Cε2 domain after each Cε3 domain from omalizumab-bound IgE is separately superimposed onto that from the 1:1 IgE-Fc/CD23 complex (see text), and displaying only omalizumab-Fv. Omalizumab heavy atoms within 2.5 Å of heavy atoms from CD23 or IgE-Cε2 are depicted as spheres.

The 1:2 IgE/omalizumab complex cannot bind to CD23 and FcεRI because the key IgE residues involved in binding both IgE receptors are bound or blocked by the two omalizumabs. The ^408^RASGK^415^ motif makes a significant free energy contribution to binding CD23, but is occupied by omalizumab. Furthermore, Glu450 and Glu452, which form hydrogen bonds with CD23:Arg188/Arg224 and CD23:His186, respectively, are locked by *intra*molecular interactions when IgE is bound by two omalizumabs (Fig. 7a). On the other hand, the ^461^PHLPR^465^ motif in the FG loop makes a large free energy contribution to binding FcεRI (Fig. 5), but these residues in both IgE chains are bound by two omalizumabs. Furthermore, the extended conformation of the Cε2 domains in the 1:2 IgE/omalizumab complex also obstructs FcεRI binding (Fig. 7b).

However, in the 1:1 IgE/omalizumab complex, can residues in the free Cε3 domain still bind CD23 or FcεRI? Since two CD23 molecules are needed to bind to IgE, binding of only one CD23 to the Cε3 domain may be too weak to compete against binding of a second omalizumab to IgE, as IgE has higher binding affinity for omalizumab than CD23^1^,^17^ (see above); hence a second omalizumab would likely outcompete CD23 for the IgE binding site. For FcεRI binding, two sets of His462 and Leu463 residues are needed. Binding of a single omalizumab to one of these sets would attenuate but not abolish IgE binding to FcεRI. So a possibility why the 1:1 IgE/omalizumab complex cannot bind FcεRI is because if the Cε2 domains were in an upright position when one omalizumab is bound (e.g., to right IgE chain in Fig. 7b with no omalizumab on the left chain), they would block FcεRI binding to the free IgE chain. On the other hand, if the Cε2 domains were bent and packed against one of the Cε3 domains, they would occlude the binding sites on one of the Cε3 domains (e.g., right IgE chain in Fig. 7c); thus omalizumab bound to the other Cε3 domain (left chain in Fig. 7c) would directly block the FcεRI-binding site.

The results herein also explain why omalizumab cannot bind FcεRI or CD23-bound IgE: Omalizumab cannot bind CD23-bound IgE because several omalizumab-binding IgE residues are bound or blocked by CD23. Soluble CD23 exists as a trimer and binds to both Cε3-4 domains using two of the three CD23 chains^17^. The two CD23 molecules bind several IgE residues in both Cε3 domains that are within the IgE/omalizumab interface; viz., Arg408, Ser411, Lys415 and Glu452. Two CD23 molecules bound to IgE would mask the ^408^RASGK^415^ epitope required for binding omalizumab, while their binding to Glu452 in both Cε3 domains might also result in conformational changes that affect binding to omalizumab (Fig. 7a). Even when one CD23 transiently dissociates from IgE, the bend between the Cε2 and Cε3 domains in free IgE, which is retained upon binding CD23^22^, obstructs omalizumab from binding to IgE. This is shown in Fig. 7d, where one of the CD23 molecules in the CD23-bound IgE structure (PDB 4gko) was omitted and the Cε2 domains (cyan) were added to this structure by superimposing the Cε3-4 domains in the free IgE structure (PDB 2 wqr) onto those in the 4gko structure, yielding a 1:1 IgE-Fc/CD23 complex. When each Cε3 domain from omalizumab-bound IgE is separately superimposed onto that from the 1:1 IgE-Fc/CD23 complex, several omalizumab heavy atoms (depicted as spheres) were found within 2.5 Å of heavy atoms from CD23 or the Cε2 domain.

In contrast to CD23, only a single FcεRI is needed to bind to IgE since it engages both Cε3 domains. Notably, FcεRI binds His462 and Leu463 from both IgE chains. Although these two residues are involved in binding omalizumab, they do not make a significant free energy contribution. Furthermore, their binding by FcεRI does not seem to obstruct omalizumab from binding to the FcεRI-free IgE chain (see Fig. 7b). However, the bend between the Cε2 and Cε3 domains in free IgE becomes more acute upon binding to FcεRI^22^ such that the Cε2 domains blocks access to the second omalizumab binding site. This is evident in Fig. 7c, which overlays the Cε3 domains in the IgE-Fc/omalizumab docked structure and the IgE-Fc/FcεRI crystal structure (PDB 2y2q) showing that the second omalizumab and the Cε2 domains from FcεRI-bound IgE would overlap.

In summary, this work offers a new understanding of how IgE interacts with omalizumab and its receptors by revealing the IgE residues critical for binding the different interacting partners of IgE. It has resolved a long-standing puzzle as to how omalizumab could prevent IgE from binding to both its receptors even when only one omalizumab binds IgE, underlining an important role of the Cε2 domains. The two nearly linear omalizumab-binding IgE epitopes found herein could potentially be used to actively induce the production of selective “omalizumab-like” anti-IgE Abs.

## Materials and Methods

### Structure determination of omalizumab

Purified omalizumab (10 mg/ml) was subjected to crystallization screening using hanging-drop vapor-diffusion method at room temperature. In general, 1 μl of omalizumab-containing solution (10 mM Tris-HCl and 100 mM NaCl pH 7.3) was mixed with 1 μl of reservoir solution in 96-well Q-Plates (Hampton Research), and equilibrated against 75 μl of the reservoir solution. These drops were set up automatically using a Mosquito Crystal crystallization robot (TTP LabTech) with 768 different reservoir conditions from Hampton Research Crystal Screen kits (Laguna Niguel, CA, USA). Optimal crystals of omalizumab were obtained from 0.1 M HEPES-Na pH 7.5, 2% v/v polyethylene glycol 400 and 2 M ammonium sulfate (mixture **A**). Prior to data collection at 100 K, the crystal was mounted in a cryoloop and soaked with mixture **A** and 30% *v*/*v* glycerol for 3 s. An X-ray diffraction dataset was collected to 2.42 Å resolution using the synchrotron radiation at beam line BL44XU at SPring-8 (Harima, Japan). The diffraction images were processed using the program *HKL*-2000^39^. The crystal structure of omalizumab was determined by molecular replacement with the program PHASER^40^ using the structures of human IgG1 Fab (PDB 1cly) and mouse CIIC1 Fab (PDB 2vl5) for the heavy chain and light chain, respectively^41^. The model building and map were further improved by computational refinement using PHENIX^42^ and COOT^43^ programs (Table 1).

### Rigid-body docking of the omalizumab-Fab and the IgE-Fc

The 2.61-Å human IgE-Fc structure (PDB 4gt7)^21^, which consists of the Cε3-Cε4 dimer in a closed conformation, was chosen for docking since it has been shown to bind omalizumab^21^. The Cε2 domains were added to this structure after superimposing the Cε3-4 domains in the IgE-Cε2-Cε3-Cε4 dimer (PDB 4j4p at 2.9 Å^20^) onto those in the 4gt7 structure. Hydrogen atoms were added using the CHARMM version 35 program and the CHARMM united-atom forcefield^44^,^45^. All Asp/Glu residues were deprotonated, Lys and Arg residues were protonated, while the histidine residues were protonated/deprotonated according to the Reduce program^27^. To eliminate steric clashes from the addition of hydrogen atoms, 100 steps of steepest descent minimization with constraints on the heavy atoms using a distance-dependent dielectric constant was performed.

The X-ray structures of omalizumab-Fab and human IgE-Fc with hydrogen atoms added were docked using the EMAP module in CHARMM^46^. We refer the reader to previous works for the EMAP methodology^46^ and its calibration on Ab/Ag complexes^23^. The Cε3 map object was generated from chain A in the 4gt7 structure with the grid spacing set to 2 Å. As only the CDR loops are involved in binding IgE, the omalizumab-Fab map object included only atoms within 8 Å of the CDR loops. A search was started at every six grid points per side for the Cε3 domain with six Fab starting rotations for each search grid point; the initial search points were restricted to be within 60^o^ of a vector between the center of the Cε3 and the center of omalizumab residues (402–415; 452–471) experimentally implicated in binding IgE. This yielded 4,180 initial orientations for omalizumab-Fab docked to the Cε3 domain. From each starting point, 50 cycles of 20 grid-threading MC minimization steps were performed and the lowest-energy conformation was scored using the EMAP-scoring functions^46^.

### Refining the rigid-body conformations using Monte-Carlo minimization

Each of the 4,180 EMAP conformations was refined using 4,000 steps of MC minimization (with a united atom force field) at 300 K allowing the entire omalizumab-Fab to be translated in any direction up to 0.3 Å or torsion angles of interface residues to be rotated by ≤30°. The torsion moves were attempted twice as often as the translation moves. The resultant structure was then minimized using adopted-basis Newton Raphson minimization for 200 steps without the electrostatic term, followed by another 300 steps including the electrostatic interaction energy computed using a distance-dependent dielectric constant.

### Identifying an IgE-Fc/omalizumab-Fab complex consistent with available experimental data

The refined Cε2-3-4/omalizumab-Fab conformations were scored by a SVM classifier^23^,^47^, as described in our previous work^23^. To determine which of the top 25 conformations from the SVM scoring was most consistent with the experimental data stated in the Results section, we created a second omalizumab-Fab by superimposing the Cε3 domains to generate a 1:2 IgE-Fc/omalizumab-Fab complex (see Fig. 4a). We also created a contact list of all IgE residues whose heavy atoms were within 5.0 Å of the heavy atoms of omalizumab residues. The top 25 SVM-ranked conformations were then grouped using MaxCluster (Structural Bioinformatics Group, Imperial College, London, 2013) with a C^α^ root-mean-square deviation (RMSD) cutoff of 4 Å. This yielded nine clusters, out of which only one containing four structures could simultaneously satisfy the available experimental data – the structure that best-fitted the experimental data was chosen (see Results).

### Generating an ensemble of conformations using MD simulations in explicit water

Although the free energy contributions of each IgE residue towards binding omalizumab, CD23, or FcεRI could be derived from a single (docked or crystal) structure, they were obtained herein from an ensemble of 8,000 conformations for each IgE complex (see next section). The conformational ensemble was generated by performing four independent MD simulations using CHARMM version37 and the CHARMM36 all-atom force field^48^ with explicit water molecules rather than running a single long simulation of each IgE complex. Since there is no experimental evidence that the Cε2 domains directly contact omalizumab, CD23, or FcεRI, they were omitted to reduce the system size. The omalizumab Cγ1 and Cl domains, which make no contacts with the IgE-Fc, were also omitted. Hence, the starting structures for the simulations were the model structure of the IgE-Fc without the Cε2 domains in complex with the omalizumab-Fv and the X-ray structures of the Cε3-4 dimer bound to FcεRI (PDB 1f6a)^15^ and CD23 (PDB 4gko, chains A, B and H)^18^. To stabilize the Cε2–Cε3 loop regions of the IgE/omalizumab-Fv complex after removing the Cε2 domains, Ala358 in both chains was mutated to Cys using PyMOL and a disulphide bridge introduced mimicking the same mutation in the IgE/FcεRI X-ray structure. The resulting systems were neutralized by adding chloride counterions at the highest electropositive locations (20, 16, and 8 Cl^–^ for the omalizumab-Fv, CD23 and IgE-FcεRI complexes, respectively) with the constraints that each counter ion was ≥6 Å from the protein surface and ≥10 Å from each other.

The neutralized system was solvated in a rectangular box containing TIP3P water molecules^49^, resulting in a total of 180,561, 180,188, and 180,321 atoms for the Cε3-4 dimer in complex with omalizumab-Fv, CD23, and FcεRI, respectively. To relieve any bad contacts in the solvated complex structure, the water molecules were subjected to rounds of minimization with constraints on the protein heavy atoms. The resulting solvated system was subjected to MD at a mean temperature of 300 K using a 1 fs time-step, periodic boundary conditions, vdW interactions switched to zero between 10 and 12 Å, and electrostatic interactions treated via the particle-mesh Ewald summation method^50^. Light constraints were placed on all backbone atoms for the first 1 ns of each simulation and then fully released. The simulations were continued for a total of 2.3 ns for the IgE/receptor complexes and 3.0 ns for IgE/omalizumab-Fv till the RMSDs of the backbone atoms of all residues within 10 Å of the other molecule from the starting structure plateaued, indicating that the system is fully equilibrated (see Supplementary Fig. S1). Coordinates were saved every 0.5 ps from the last 1 ns of each simulation.

### Computing the binding free energy and per-residue contributions

For each of the 8,000 conformations generated by the four independent simulations of each IgE complex, water molecules and counter ions were removed and the gas-phase electrostatic interaction energy (Δ*E*_*gas*_^*elec*^) between the Cε3-4 dimer and its interacting partner (denoted by X) was calculated with a 14 Å cut-off. The Δ*E*_*gas*_^*elec*^ values were sorted into 25 evenly distributed Δ*E*_*gas*_^*elec*^ clusters. Within each cluster, the conformation whose gas-phase electrostatic interaction energy is closest to the mean of the cluster was chosen as the representative to compute the free energy of X binding to IgE in solution. Using the following thermodynamic cycle,


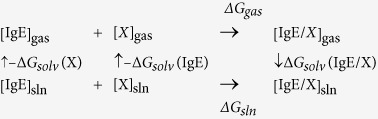


the binding free energy in aqueous solution, Δ*G*_*sln*_, is given by,


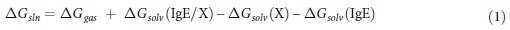


ΔG_sln_ was computed using the popular Molecular Mechanics Poisson-Boltzmann Surface Area (MM-PBSA) approach^51^, based on the following approximations. It was derived from MD trajectories of the complex because no large structural changes upon binding are expected, as the Cε3 domains in the crystal structures of free and receptor-bound IgE align well with C^α^ RMSDs ~ 1 Å. Following previous studies^52^,^53^, the gas-phase binding free energy, Δ*G*_*gas*_, was approximated as a sum of the vdW and electrostatic binding energies computed with the same force field employed in the simulations using a cutoff of 999 Å, while the solvation free energy, Δ*G*_*solv*_, was approximated as a sum of the electrostatic (Δ*G*_*solv*_^*elec*^) and nonelectrostatic (Δ*G*_*solv*_^*nonel*^) contributions. The Δ*G*_*solv*_^*elec*^ was estimated by finite–difference solution of the linearized Poisson–Boltzmann equation implemented in the APBS program^25^ using a dielectric constant of 1 for the protein and 78.54 for the solvent. The APBS calculations were performed using a cubic grid (321 points per side) with an initial grid spacing of 1.5 Å, which was successively decreased to 1.0 and 0.5 Å. Charges were mapped onto the nearest and next-nearest neighbor grid points using a cubic B-spline discretization, while the dielectric boundary was described by a cubic-spline surface^54^. The Δ*G*_*solv*_^*nonel*^ was estimated by *γ*×SASA, where *γ*  = 8 cal/mol/Å^2^. Since the different clusters contain different numbers of conformations, the Δ*G*_*sln*_ free energies derived from the representative conformations were weighted according to the number of structures in each cluster to give an average binding free energy for that simulation. The results from all four simulations for each complex were then averaged to give a final Δ*G*_*sln*_ and the respective standard deviation.

The contribution of an individual residue *i* to the binding free energy, Δ*G*_*sln*_(*i*), can be determined from (a) the pairwise vdW and electrostatic interactions of residue *i* in each IgE complex, (b) *G*_*solv*_^*nonel*^(*i*) = *γ* × SASA(*i*), where SASA(*i*) is the SASA of residue *i* in the free or bound protein, and (c) *G*_*solv*_^*elec*^(*i*), which can be computed by summing over all charges, the product of the charge and the potential at the position of the charge due to the atomic charges from residue *i* in the protein. For residue *i* in each complex, the four sets of weighted per-residue free energies were used to calculate the average Δ*G*_*sln*_(*i*) and corresponding standard deviation (see Tables S1, S3a, and S3b).

## Additional Information

**How to cite this article**: Wright, J. D. *et al*. Structural and Physical Basis for Anti-IgE Therapy. *Sci. Rep*. **5**, 11581; doi: 10.1038/srep11581 (2015).

## Supplementary Material

Supplementary Information

## Figures and Tables

**Figure 1:**
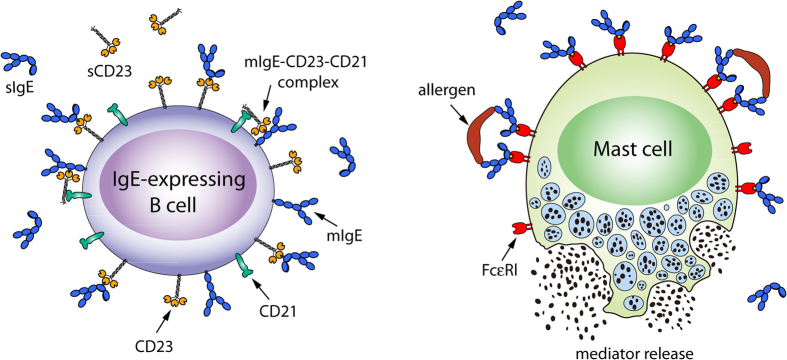
How IgE mediates an allergic reaction via interaction with its two receptors. (**Left**) Interactions of membrane-bound IgE (mIgE, blue) with CD23 (tangerine) on B-cells regulates soluble IgE (sIgE) production. (**Right**) Cross-linking of IgE bound to FcεRI (scarlet) on mast cells or basophils by allergens (brown) triggers the release of mediators, causing allergy.

**Table 1:** Summary of data processing and refinement statistics for the solution of the X-ray crystal structure of the omalizumab Fab.

Name	Omalizumab
PDB code	2XA8
***Data collection***	
Resolution (Å)	30.00 - 2.41 (2.50 – 2.41)
Space group	*P*2_1_2_1_2_1_
Unit-cell	
*a* (Å)	64.60
*b* (Å)	73.85
*c* (Å)	141.13
α (°)	90.00
β (°)	90.00
γ (°)	90.00
No. of reflections	
Measured	124171
Unique	25869
Completeness (%)	98.6 (94.1)
*R*_merge_ (%)^a^	10.0 (82.0)
Mean *I*/*σ*(*I*)	14.6 (1.4)
Multiplicity	4.8 (4.1)
***Refinement***	
No. reflections used	25778 (2553)
*R*_*work*_ (%)	22.3 (39.5)
*R*_*free*_ (%)	26.9 (47.0)
Geometry deviations	
Bond lengths (Å)	0.006
Bond angles (°)	0.98
No. of atoms / Mean	
B-values (Å^2^)	
Protein atoms	3325 / 44.7
Water molecules	72 / 38.7
Ramachandran plot (%)	
Most favored	88.3
Additionally allowed	11.2
Disallowed	0.5

Values in parentheses are for the highest resolution shell.

^a^