# Supplementary material for: Structural and Physical Basis for Anti-IgE Therapy
Source: Sci Rep. 2015 Jun 26;5:11581. doi: 10.1038/srep11581 (PMC4481376; doi:10.1038/srep11581)
Supplement: Supplementary Information [file srep11581-s1.pdf]

Structural and Physical Basis for Anti-IgE Therapy

Jon D. Wright[1,2], Hsing-Mao Chu[2], Chun-Hsiang Huang[2], Che Ma[2], Tse Wen Chang[2,*] and Carmay Lim[1,3*]

[1]Institute of Biomedical Sciences, Academia Sinica, Taipei 115, Taiwan, [2]The Genomics Research Center, Academia Sinica 115, Taiwan, and [3]Department of Chemistry, National Tsing Hua University, Hsinchu 300, Taiwan

*Correspondence to: Carmay Lim, Institute of Biomedical Sciences, Academia Sinica, Taipei 115, Taiwan. E-mail: carmay@gate.sinica.edu.tw or Tse Wen Chang, Genomics Research Center, Academia Sinica 115, Taiwan. E-mail: twchang@gate.sinica.edu.tw

Supplementary Table 1. Binding free energy contributions from the IgE-Cε3-4/omalizumab-Fv interface residues.

Residue *i* [a]	Structure	$\Delta G_{sln}(i)$ [b]	Hydrogen bonds [c]	vdW contacts [d]
A:Pro364	Cε2-3 loop	–0.6±0.9		
B:Arg365	Cε2-3 loop	–2.4±1.4	BB---A:Asn363 BB	
		–3.0±1.1		
B:Asn430	loop DE	–2.0±2.0	SC---B:Thr434 BB<br>SC---B:Thr434 SC	
		–2.0±2.0		
A:Thr405	β-strand C	–2.7±1.9	BB---A:Arg457 BB<br>BB---A:Arg457 BB	
A:Ser407	β-strand C	**–4.9±5.2**	SC---L3:Asp94 SC[d]<br>BB---A:Gln455 BB<br>BB---A:Gln455 BB	L3:Glu93 SC[e]
A:Arg408	β-strand C	–1.0±1.2	BB---A:Gly414 BB<br>SC---A:Lys415 BB<br>SC---A:Pro416 BB<br>SC---A:Asp447 SC<br>SC---A:Glu452 SC	
**A:Ala410**	loop CD	**–3.1±0.8**	BB---A:Thr453 BB	H3:Trp100B
A:Ser411	loop CD	**–3.3±0.6**	SC---A:Glu452 SC	H2:Asn58
A:Gly414	loop CD	–1.8±0.3	BB---A:Arg408 BB	H2:Asn58
A:Lys415	loop CD	**–3.2±0.9**	SC---H2:Asp54 SC<br>BB---A:Arg408 SC<br>SC---A:Glu452 SC	H2:Ser56
A:Pro416	loop CD	–0.6±0.6	BB---A:Arg408 SC	
		**–17.8±1.4**		
A:Glu450	helix B	–3.0±2.5	BB---A:Arg446 BB<br>SC---A:Arg446 SC	H2:Tyr53
A:Gly451	EF' turn	–0.9±1.1		H2:Tyr53
A:Glu452	EF' turn	–0.7±1.2	SC---A:Arg408 SC<br>SC---A:Ser411 SC<br>BB---A:Asp447 BB<br>BB---A:Thr473 SC	
A:Thr453	EF' turn	–0.6±0.9	BB---A:Ala410 BB	
A:Gln455	β-strand F	–1.7±0.6	BB---A:Ser407 BB<br>BB---A:Ser407 BB	
A:Arg457	β-strand F	**–7.7±1.2**	SC---L3:His92 SC<br>SC---L3:Glu93 SC<br>BB---A:Thr405 BB<br>BB---A:Thr405 BB	L:Ile2
A:Thr459	β-strand F	–2.3±1.3	BB---A:Asn403 BB<br>BB---A:Asn403 BB	
A:His460	loop FG	–0.1±0.2	BB---A:Leu463 BB	
A:Pro461	loop FG	–0.3±0.4		
A:His462	loop FG	–0.5±0.5		
A:Leu463	loop FG	–0.3±0.1	BB---A:His460 BB	L1:Gln27

A:Pro464	loop FG	–1.5±0.9	BB---L1:Ser27A BB	L1:Gln27
A:Arg465	loop FG	**–8.3±2.5**	SC---L1:Asp27C SC<br>BB---A:Arg365 BB<br>BB---A:Gly366 BB	L1:Ser27A<br>L:Gly68
A:Ala466	loop FG	–1.7±0.7	BB---L1:Ser27A BB	L1:Val27B<br>L1:Asp27C<br>L3:His92
A:Leu467	β-strand G	–2.9±0.3	BB---A:Val458 BB	L1:Asp27C
A:Met469	β-strand G	**–5.0±0.5**	BB---L1:Asp27C BB	L1:Tyr27D<br>L1:Tyr32<br>L3:His92
**A:Arg470**	β-strand G	**–3.4±0.6**	SC---L1:Asp28 SC<br>SC---A:Ala369 BB<br>BB---A:Cys456 BB<br>BB---A:Cys456 BB	
A:Ser471	β-strand G	–0.8±0.8	SC---L1:Tyr27D SC	
		**–41.5±0.9**		
H1:Tyr33	CDR H1	–1.2±0.7		
		–1.2±0.7		
H2:Ser50	CDR H2	–0.3±0.5		
H2:Thr52	CDR H2	–0.6±0.4		
H2:Tyr53	CDR H2	**–5.1±2.2**		A:Glu450<br>A:Gly451
H2:Asp54	CDR H2	–0.3±0.9	SC---A:Lys415 SC	A:Lys415
H2:Ser56	CDR H2	–1.5±0.6		A:Lys415
H2:Thr57	CDR H2	–0.7±0.2		
H2:Asn58	CDR H2	–1.5±0.3		A:Ser411<br>A:Gly414
		**–9.8±0.7**		
H3:His97	CDR H3	–0.1±0.3		
H3:His100A	CDR H3	–1.4±0.7		
H3:Trp100B	CDR H3	–1.5±0.7		A:Ala410
		–3.0±0.6		
L:Ile2		–0.5±0.1		A:Arg457
		–0.5±0.1		
L1:Gln27	CDR L1	**–3.7±2.3**		A:Pro464
L1:Ser27A	CDR L1	–2.5±0.9	BB---Pro464 BB<br>BB---A:Ala466 BB	A:Arg465
L1:Val27B	CDR L1	–2.4±1.5		A:Ala466
L1:Asp27C	CDR L1	–2.8±1.2	SC---A:Arg465 SC<br>BB---A:Met469 BB	A:Leu467<br>A:Met469
L1:Tyr27D	CDR L1	**–4.9±1.7**	SC---A:Ser471 SC	A:Met469
L1:Asp28	CDR L1	–2.3±1.3	SC---A:Arg470 SC	
L1:Ser31	CDR L1	–0.3±0.4		
L1:Tyr32	CDR L1	–3.0±0.4		A:Met469
		**–21.9±1.3**		
L:Ser67		–1.1±1.0		
L:Gly68		–1.2±0.7		A:Arg465
L:Thr69		–1.0±1.1		

L:Phe71		–0.2±0.2		
		**–3.5±0.8**		
L3:Ser91	CDR L3	0.1±0.6		
L3:His92	CDR L3	**–5.1±2.5**	SC---A:Arg457 SC	A:Ala466<br>A:Met469
L3:Glu93	CDR L3	**–5.3±2.0**	SC---A:Arg457 SC	
L3:Asp94	CDR L3	**–4.5±5.5**		
L3:Tyr96	CDR L3	**–3.2±1.8**		
		**–18.1±2.5**		

[a]Residues with interaction free energies exceeding 3 kcal/mol are highlighted in bold; those that are experimentally implicated in binding omalizumab are highlighted in *italics*.
[b]Absolute interaction free energies exceeding 3 kcal/mol are highlighted in bold; the per-residue free energies for each region are summed up in the shaded row.
[c]See text for definition of hydrogen bond and vdW contacts.
[d]The Ser407 side chain was within 5 Å of the L3:Asp94 side chain in two simulations formed a hydrogen bond in one of the simulations, and was in vdW contact with the L3:Glu93 side chain in another simulation.

Supplementary Table 2: High and low affinity receptor hydrogen bonds observed in the X-ray crystal structure and during the sampling phase of the MD trajectories.[a]

IgE atom *i*	Receptor atom *j*[b]	R_{ij}, Å (X-ray)	R_{ij}, Å (MD1)	R_{ij}, Å (MD2)	R_{ij}, Å (MD3)	R_{ij}, Å (MD4)
A:Arg408 N^{H1}	CD23:Tyr189 O^{H}	3.2	3.1	3.0	3.7	3.0
A:Lys415 N^{Z}	CD23:Asp193 O^{D1}	2.8	2.9	2.8	3.0	2.9
A:Asp447 O^{D1}	CD23:Arg188 N^{H2}	3.0	*7.4*	*4.6*	*5.8*	*4.6*
A:Asp447 O^{D1}	CD23:Tyr189 O^{H}	3.0	3.7	*6.7*	3.6	*5.6*
A:Glu450 O^{E1}	CD23:Arg188 N^{E}	3.0	2.9	3.0	2.9	2.9
A:Glu450 O^{E1}	CD23:Arg188 N^{H2}	3.1	2.7	2.9	2.9	3.0
A:Glu450 O^{E2}	CD23:Arg224 N^{H1}	3.0	2.8	4.0	2.8	*6.8*
A:Glu452 O^{E1}	CD23:His186 N^{E2}	3.2	2.9	2.9	2.9	2.9
A:Arg499 N^{E}	CD23:Ser254 O^{G}	2.8	*5.3*	*5.0*	*5.0*	*5.4*
A:Arg499 N^{H2}	CD23:Ser254 O^{G}	2.6	*5.6*	*4.1*	*4.0*	*4.7*
A:Arg499 N	CD23:Gln255 O	3.0	3.1	3.1	3.4	3.0
A:Arg499 N^{H1}	CD23:Asp258 O^{D2}	2.8	2.8	3.1	3.2	2.7
A:Arg499 N^{H2}	CD23:Asp258 O^{D1}	2.9	3.1	3.2	3.2	2.7
A:Gln598 N^{E2}	CD23:Asp227 O	3.2	4.2	3.8	3.6	3.6
A:Asn363 O	FcεRI:Gln157 N^{E2}	3.5	3.1	*6.1*	3.4	3.4
A:Arg365 N^{H1}	FcεRI:Gln157 O^{E1}	3.2	3.0	*4.9*	*6.5*	*7.1*
A:Gly366 N	FcεRI:Trp156 O	3.2	2.9	3.0	2.9	2.9
B:Arg365 N^{H1}	FcεRI:Glu132 O^{E1}	2.4	*3.6*	3.4	3.3	*4.2*
B:Gly366 O	FcεRI:Lys117 N^{Z}	3.2	3.7	3.8	3.8	2.9
B:Asp394 O^{D2}	FcεRI:Lys117 N^{Z}	3.4	2.8	2.7	2.7	2.7
B:Asp394 O	FcεRI:Tyr129 O^{H}	2.8	3.0	2.8	2.8	2.8
B:Ala396 N	FcεRI:Tyr131 O^{H}	3.3	3.1	3.1	3.1	3.1
B:His462 N^{D1}	FcεRI:Tyr131 O^{H}	2.6	*7.2*	*5.9*	*4.7*	*6.2*

[a]Distances that deviate > 1 Å from the respective X-ray distances are highlighted in italics.
[b]For CD23, chain H was used.

Supplementary Table 3a. Binding free energy contributions from the interface residues upon IgE-Cε3-4 binding to its low–affinity receptor, CD23.

Residue *i*[a]	Structure	ΔG_{sln}(*i*)[b]	Hydrogen bonds[c]	vdW contacts[d]
A:Phe381	α–helix A	–1.6±0.3	BB---Pro376 BB	CD23:Asp227<br>CD23:Leu228
A:Lys384	AB′ turn	–0.6±0.2		
		–2.2		
A:Arg408	β–strand C	–1.2±0.5	BB---Gly414 BB<br>SC---Lys415 BB<br>SC---Pro416 BB	CD23:Tyr189
A:Ala410	CD loop	–0.9±0.2	BB---Thr453 BB	
A:Ser411	CD loop	–1.8±0.2	BB---Glu452 SC<br>SC---Glu452 SC	CD23:His186<br>CD23:Tyr189
A:Lys415	CD loop	**–3.7±0.5**	BB---Arg408 SC<br>SC---CD23:Asp193 SC	
		**–7.6**		
A:Arg446	α–helix B	–2.2±0.5	BB---Glu450 BB	CD23:Leu226
**A:Asp447**	α–helix B	**–3.5±1.4**	BB---Glu452 BB	CD23:Tyr189
A:Ile449	α–helix B	–3.0±0.3		CD23:Leu226<br>CD23:Asp227
**A:Glu450**	α–helix B	**–3.9±0.6**	BB---Arg446 BB<br>SC---CD23:Arg188 SC<br>SC---CD23:Arg224 SC	CD23:Trp184<br>CD23:Val185<br>CD23:Cys273
A:Gly451	EF′ turn	–1.6±0.2		CD23:Gln183<br>CD23:Val185
A:Glu452	EF′ turn	1.0±0.4	SC---Ser411 BB<br>SC---Ser411 SC<br>BB---Asp447 BB<br>SC---Thr453 BB<br>BB---Thr473 SC<br>SC---CD23:His186 SC	CD23:Val185<br>CD23:Tyr189
A:Tyr454	β–strand F	–0.1±0.3	BB---Thr472 BB<br>BB---Thr472 BB	
		**–13.3**		
**A:Lys474**	Cε3–Cε4 loop	**–4.5±1.0**	SC---Glu592 SC<br>SC---CD23:Asp227 SC	
**A:Ser476**	Cε3–Cε4 loop	**–9.2±1.2**	SC---CD23:Asp274 SC	CD23:Phe272
A:Gly497	Cε4	–0.9±0.2		CD23:Phe272
A:Pro498	Cε4	–1.5±0.4		CD23:Gln255
**A:Arg499**	Cε4	**–7.7±0.8**	SC---Glu592 SC<br>SC---CD23:Asp227 SC<br>SC---CD23:Gln255 BB<br>BB---CD23:Gln255 BB<br>SC---CD23:Asp258 SC	CD23:Ser254<br>CD23:Gly256
A:Ala500	Cε4	–0.6±0.2	BB---Met530 BB<br>BB---Met530 BB	
A:Ala501	Cε4	–0.3±0.1		
		**–24.7**		

A:Glu592	Cε4	1.0±0.7	SC---Lys474 SC<br>SC---Arg499 SC<br>BB---Gln598 SC	CD23:Asp227
A:Ala593	Cε4	–1.2±0.8	BB---His591 SC	CD23:Ser254
A:Ala594	Cε4	–0.2±0.3		
A:Ser595	Cε4	–0.5±0.5		
A:Ser597	Cε4	–0.9±0.6		CD23:Leu228
A:Gln598	Cε4	–1.8±0.7	BB---A:His591 BB<br>SC---A:Glu592 BB	CD23:Asp227<br>CD23:Leu228
		**–3.6**		
CD23:Gln183		–1.3±0.3		A:Gly451
CD23:Trp184		–1.2±0.1		A:Glu450
**CD23:Val185**		**–3.2±0.2**		A:Glu450<br>A:Gly451<br>A:Glu452
CD23:His186		–0.8±0.5	SC---A:Glu452 SC	A:Ser411
**CD23:Arg188**		**–3.5±0.6**	SC---A:Glu450 SC	
**CD23:Tyr189**		**–5.7±1.3**		A:Arg408<br>A:Ser411<br>A:Asp447<br>A:Glu452
CD23:Asp193		–0.1±0.4	SC---A:Lys415 SC	
CD23:Arg224		–1.4±0.3	SC---A:Glu450 SC	
CD23:Asn225		–0.5±0.2		
CD23:Leu226		–2.9±0.3		A:Arg446<br>A:Ile449
CD23:Asp227		1.1±0.5	SC---A:Lys474 SC<br>SC---A:Arg499 SC	A:Phe381<br>A:Ile449<br>A:Glu592<br>A:Gln598
**CD23:Leu228**		**–4.3±0.9**		A:Phe381<br>A:Gln598
CD23:Lys229		–0.6±0.6		
CD23:Gly230		–0.4±0.1		
CD23:Arg253		–0.1±0.5		
CD23:Ser254		–3.0±2.5		A:Arg499<br>A:Ala593
**CD23:Gln255**		**–3.1±0.4**	BB---A:Arg499 SC<br>BB---A:Arg499 BB	A:Pro498
CD23:Gly256		–2.4±0.3		A:Arg499
CD23:Glu257		–0.7±0.4		
CD23:Asp258		1.4±0.8	SC---A:Arg499 SC	
CD23:Phe272		–2.4±0.4		A:Ser476<br>A:Gly497
CD23:Cys273		–1.2±0.2		A:Glu450
**CD23:Asp274**		**–9.7±1.0**	SC---A:Ser476 SC	

[a]Residues with interaction free energies exceeding 3 kcal/mol are highlighted in bold; those that are experimentally implicated in binding omalizumab are highlighted in *italics*.

[b]Absolute interaction free energies exceeding 3 kcal/mol are highlighted in bold; the per-residue free energies for each region are summed up in the shaded row.
[c]See text for definition of hydrogen bond and vdW contacts.

Supplementary Table 3b. Binding free energy contributions from the interface residues upon IgE-Cε3-4 binding to its high–affinity receptor, FcεRI.

Residue *i*[a]	Structure	$\Delta G_{sln}(i)$[b]	Hydrogen Bonds[c]	Vdw Contacts[d]
A:Asp361	Cε2–Cε3 loop	0.1±0.3		
A:Asn363	Cε2–Cε3 loop	–2.0±1.1		FcεRI:Gln157
A:Pro364	Cε2–Cε3 loop	–1.6±0.3		FcεRI:Trp156, Gln157
**A:Arg365**	Cε2–Cε3 loop	**–4.6±0.6**	SC---A:Asp394 SC	FcεRI:Trp156, Gln157
A:Gly366	Cε2–Cε3 loop	–2.2±0.1	BB---FcεRI:Trp156 BB	FcεRI:Leu158
A:Val367	Cε2–Cε3 loop	–0.7±0.1		FcεRI:Leu158
A:Ser368	β–strand A	–0.1±0.2	BB---A:Val393 BB<br>BB---A:Val393 BB	
		**–11.1**		
**B:Arg365**	Cε2–Cε3 loop	**–3.5±0.7**	SC---B:His462 BB<br>SC---FcεRI:Glu132 SC	FcεRI:Tyr131
B:Gly366	Cε2–Cε3 loop	–0.6±0.2	BB---B:Asn363 BB	FcεRI:Lys117
B:Val367	Cε2–Cε3 loop	–0.7±0.2	BB---B:Pro364 BB	FcεRI:Tyr131z
		**–4.9**		
A:Pro461	FG loop	–0.4±0.0		
A:His462	FG loop	–2.5±1.1	BB---His460 SC<br>BB---FcεRI:Trp110 SC	FcεRI:Trp113
A:Leu463	FG loop	–2.0±0.6		FcεRI:Trp87
A:Pro464	FG loop	**–8.9±1.2**	BB---FcεRI:Ser85 SC	FcεRI:Asp86, Trp87
A:Arg465	FG loop	**–3.2±0.2**	SC---FcεRI:Asp86 SC	FcεRI:Trp87
		**–17.0**		
B:His460	FG loop	–0.5±0.1	SC---B:His462 BB<br>BB---B:Leu463 BB	
B:His462	FG loop	–1.1±0.3	BB---B:Arg365 SC<br>BB---B:His460 SC	FcεRI:Trp130
B:Leu463	FG loop	–0.5±0.3	BB---B:His460 BB	
		**–2.1**		
**B:Asp394**	β–strand B	**–4.2±0.4**	SC--- FcεRI:Lys117 SC<br>BB---FcεRI:Tyr129 SC	FcεRI:Tyr131
B:Leu395	β–strand B	–2.1±0.1	BB---B:Leu435 BB	FcεRI:Tyr129, Tyr131
**B:Ala396**	BC loop	**–3.4±0.1**	BB---FcεRI:Tyr131 SC	FcεRI:Tyr129, Trp130
B:Pro397	BC loop	–1.5±0.3		
B:Ser398	BC loop	–0.4±0.3	SC---B:Gly400 BB	
		**–11.6**		
B:Arg428	DE loop	–1.6±0.5		
B:Asn430	DE loop	–2.6±0.1	SC---B:Thr434 SC<br>SC---B:Thr434 BB	FcεRI:Ile119, Tyr121, Ala126
B:Gly433	DE loop	–1.2±0.3	BB---B:Gln427 BB	
B:Thr434	DE loop	–1.2±0.2	BB---B:Gln427 BB<br>SC---B:Asn430 SC<br>BB---B:Asn420 SC	FcεRI:Tyr129
		**–6.5**		
FcεRI:Ser85		–0.7±1.7	SC---A:Pro464 BB	
FcεRI:Asp86		–0.1±0.3	SC---A:Arg465 SC	A:Pro464
**FcεRI:Trp87**		**–4.9±0.7**		A:Leu463, Pro464, Arg465
**FcεRI:Trp110**		**–3.2±0.6**	SC---A:His462 BB	A:Pro464
FcεRI:Arg111		–1.8±1.9		
FcεRI:Trp113		–1.6±0.2		A:His462

FcεRI:Tyr116		–0.6±0.8		
**FcεRI:Lys117**		**–3.1±0.8**	SC---B:Asp394 SC	B:Gly366, Asp394
FcεRI:Ile119		–1.5±0.1		B:Asn430
FcεRI:Tyr121		0.0±0.8		B:Asn430
FcεRI:Gly124		–0.2±0.1		
FcεRI:Glu125		–0.6±0.4		
FcεRI:Ala126		–1.4±0.2		B:Asn430
**FcεRI:Tyr129**		**–8.6±0.9**	SC---B:Asp394 BB	B:Ala396, Leu395, Thr434
FcεRI:Trp130		–2.9±0.1		B:Ala396, His462
FcεRI:Tyr131		–2.8±1.1	SC---B:Ala396 BB	B:Arg365, Val367, Asp394, Leu395
FcεRI:Glu132		0.4±0.9	SC---B:Arg365 SC	
**FcεRI:Trp156**		**–4.3±1.1**	BB---A:Gly366 BB	**A**:Pro364, Arg365
**FcεRI:Gln157**		**–3.9±1.5**		A:Asn363, Pro464, Arg365
**FcεRI:Leu158**		**–3.3±0.4**		A:Gly366, Val367
FcεRI:Tyr160		–0.3±0.4		

[a]Residues with interaction free energies exceeding 3 kcal/mol are highlighted in bold; those that are experimentally implicated in binding omalizumab are highlighted in *italics*.
[b]Absolute interaction free energies exceeding 3 kcal/mol are highlighted in bold; the per-residue free energies for each region are summed up in the shaded row.
[c]See text for definition of hydrogen bond and vdW contacts.

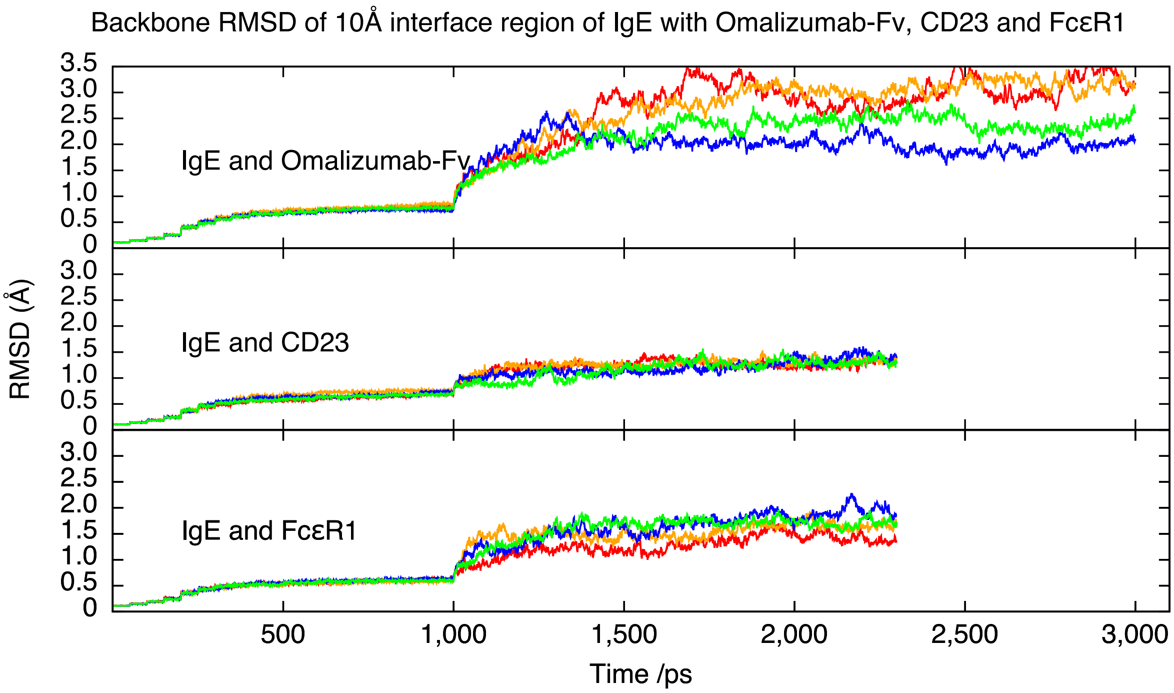

Supplementary Figure S1. Rmsd plot for each simulation for the backbone of all residues within 10Å of the other molecule.
